# Planococcus notacanthi sp. nov., isolated from the skin of a deep-sea snub-nosed spiny eel

**DOI:** 10.1099/ijsem.0.006298

**Published:** 2024-03-21

**Authors:** Shona Uniacke-Lowe, Catherine Stanton, Colin Hill, Paul Ross

**Affiliations:** 1 School of Microbiology, University College Cork, Cork, Ireland; 2 APC Microbiome Ireland, Cork, Ireland; 3 Teagasc Food Research Centre, Fermoy, Ireland

**Keywords:** *Planococcus*, *Caryophanaceae*, marine fish, deep-sea, novel species, biosynthetic gene clusters

## Abstract

A novel bacterial strain, APC 4016^T^, was previously isolated from the skin of a snub-nosed spiny eel, *Notacanthus chemnitzii*, from a depth of 1000 m in the northern Atlantic Ocean. Cells were aerobic, cocci, motile, Gram-positive to Gram-variable staining, and gave rise to orange-pigmented colonies. Growth occurred at 4–40 °C (optimum, 25–28 °C), pH 5.5–12 (optimum, pH 7–7.5), and 0–12 % (w/v) NaCl (optimum, 1 %). 16S rRNA gene phylogenetic analysis confirmed that strain APC 4016^T^ belonged to the genus *Planococcus* and was most closely related to *Planococcus okeanokoites* IFO 12536^T^ (98.98 % 16S similarity). However, digital DNA–DNA hybridization and average nucleotide identity values between these two strains were low, at 20.1 and 83.8 %, respectively. Major (>10 %) cellular fatty acids of strain APC 4016^T^ were iso-C_14 : 0_, anteiso-C_15 : 0_ and C_16 : 1_-ω-Alc. The predominant respiratory quinones were menaquinones 5, 6, 7 and 8. The major cellular polar lipids were phosphatidylglycerol, diphosphatidylglycerol and phosphatidylethanolamine, and three unknown lipids were also present. The draft genome sequence is 3.6 Mb with a G+C content of 45.25 mol%. This strain was previously shown to have antimicrobial activity and to encode bacteriocin and secondary metabolite biosynthetic gene clusters. Based on the phylogenetic analysis and its distinct phenotypic characteristics, strain APC 4016^T^ is deemed to represent a novel species of the genus *Planococcus*, and for which the name *Planococcus notacanthi* sp. nov. is proposed. The type strain of this species is APC 4016^T^ (=DSM 115753^T^=NCIMB 15463^T^).

## Background

The genus *Planococcus* was first proposed by Migula [1] in order to distinguish motile marine cocci from the genus *Micrococcus*. It has proven to be a difficult taxonomic group to describe; the genus has undergone several emendations [2,3] and members have been frequently reclassified, mainly between *Planococcus* and *Planomicrobium* [4,6]. Previously, members of the genera *Planococcus* and *Planomicrobium* were primarily distinguished based on signature nucleotides of the 16S rRNA gene sequence, particularly those at positions 183 and 190 [7]. However, an extensive demarcation of *Planococcaceae* and related families was recently proposed by Gupta and Patel [8]. They carried out extensive phylogenetic and comparative analysis of core protein sequences from 124 complete genomes from *Caryophanaceae*, *Planococcaceae* and selected *Bacillaceae* species. From their analysis, they proposed the consolidation of the families *Planococcaceae* and *Caryophanaceae* (under the name *Caryophanaceae*). They established that sequences from each species could be reliably grouped into distinctive clades, which were distinguished based on their phylogenetic grouping and on the presence of unique molecular markers (‘conserved signature indels’). These conserved signature indels were specific to either *Caryophanaceae*/*Planococcaceae* members or to clades within this family. They also proposed the creation of a novel genus, *Metaplanococcus*, the reclassification of several species, and that the genera *Planococcus* and *Planomicrobium* be united under the name ‘*Planococcus’*. The emended genus *Planococcus* now includes most species from the genus *Planomicrobium*. To date, there are 26 valid type strains within the genus *Planococcus* (*Planococcus*/*Planomicrobium*). Species have been isolated from a wide variety of sources, primarily marine or halophilic environments, such as marine sediment [3,5], algal mats [9,10] and seafood [4,11]. Others have been found in extreme conditions including sub-zero temperatures [12,14], deep-sea sediment [15] and in polluted soils [16,17]. Members of the genus *Planococcus* are of great biotechnological interest because of their robustness and diverse biological activities, such as production of cold-adapted and halotolerant enzymes [18,20], radiation tolerance [21], plant growth promotion [22] and antioxidant and cytotoxic activities [23,24]. They have also been extensively studied for their potential applications in bioremediation and polysaccharide degradation [25,28].

Strain APC 4016^T^ was previously isolated during a study of antimicrobial-producing isolates from the microbiome of deep-sea fish, and subsequently shown to encode secondary metabolites and bacteriocins [29]. In this study, we report the taxonomic characterization of a *Planococcus* bacterial strain, APC 4016^T^, isolated from the skin of a deep-sea snub-nosed spiny eel (*Notacanthus chemnitzii*).

## Methods

### Isolate information

Strain APC 4016^T^ was previously isolated from the skin of a snub-nosed spiny eel (*Notacanthus chemnitzii*) [29]. In brief, the fish was collected by research vessels from a depth of approximately 1000 m in international waters near the Grand Banks of Newfoundland in the north-western Atlantic Ocean (43.282 N 49.121 W) [30]. Swabs were taken of the skin and plated onto BD Difco marine agar 2216 (MA) and incubated aerobically for 3 weeks at 4 °C. An orange-coloured colony was selected and purified through sub-culturing on MA. The strain, designated APC 4016, was deposited into the APC Culture Collection, cells of which were preserved in 35 % (v/v) glycerol suspensions at −80 °C [29]. This strain has also been deposited into the DSMZ-German Collection of Microorganisms and Cell Cultures GmbH (=DSM 115753^T^) and the National Collection of Industrial, Food and Marine Bacteria (= NCIMB 15463^T^).

### Genome sequencing, assembly and annotation

Strain APC 4016^T^ was cultured in BD Difco marine broth 2216 (MB) for 72 h at 20 °C. Bacterial genomic DNA (gDNA) was extracted and sequenced, as previously described [29]. Briefly, gDNA was extracted using the GeneJET Genomic DNA Purification Kit (Thermo Scientific) and sequenced by MicrobesNG (https://microbesng.com/; University of Birmingham, UK). The draft genome was assembled using SPAdes (*de novo* assembly method), and assembly quality was assessed using quast. The assembled contigs were submitted to GenBank and annotated upon submission using NCBI’s Prokaryotic Annotation Pipeline. For this study, the draft assembly was annotated using prokka [31]. Additionally, functional subsystems were predicted using RASTtk (version 2.0) and seed [32,33]. AntiSMASH and bagel4 were used to screen for the presence of secondary metabolite and bacteriocin biosynthetic gene clusters (BGCs), respectively, as previously reported [29].

### 16S rRNA gene phylogeny

The compete 16S rRNA gene sequence (1548 bp) was extracted from the gDNA sequence data using Barrnap (version 0.9; https://github.com/tseemann/barrnap). The 16S sequence was then queried using the 16S-based ID application on the EzBioCloud server [34]. Sequence files (.fasta) of the top hits were downloaded and imported into mega X [35]. Multiple alignments with the query sequence were created using ClustalW [36] within the mega X program. Phylogenetic trees (neighbour-joining [37], maximum-likelihood [38] and maximum-parsimony [39]) were inferred using the Kimura two-parameter method [40] and bootstrap analyses was based on 1000 replicates [41]. The tree was rooted by including sequences from the family *Caryophanaceae* (*Caryophanaceae*/*Planococcaceae*) as outliers; namely, the 16S rRNA gene sequences from *Sporosarcina saromensis* HG645 (AB243859) and *Psychrobacillus lasiicapitis* NEAU-3TGS17 (KP219721).

### Genomic phylogeny

For the following analyses, all available *Planococcus*/*Planomicrobium* valid type strain reference genomes (*n*=22) were downloaded from the GenBank (www.ncbi.nlm.nih.gov/data-hub/genome/) and JGI (https://img.jgi.doe.gov/) databases. Assemblies were annotated using prokka to obtain the GFF3 files for subsequent analysis. Pairwise average nucleotide identity (ANI) values were calculated between APC 4016^T^ and the *Planococcus*/*Planomicrobium* type strain reference assemblies with Pyani (version 0.2.12; [42]) using the ANI MUMer (ANIm) method [43]. roary [44] was used to generate multiple sequence alignments of the core genes of APC 4016^T^ and the type strain reference genomes. The annotated genome of *Sporosarcina aquimarina* S/N-308-OC-B4 was included as an outlier (roary parameters: 95 % ‘identity for blast’, 95 % ‘percentage of isolates a gene must be in to be core’, 100 000 cluster limit). RaxML [45] was used to generate a maximum-likelihood phylogenetic tree from the core gene alignment, which was then visualized in mega X. Additionally, the DSMZ Type (Strain) Genome Server (TYGS) annotation platform [46] was used to calculate digital DNA–DNA hybridization (dDDH) values between APC 4016^T^ and related type strains from the database.

### Biochemical and phenotypic characterisation

Colony morphology was assessed by incubating aerobically at 25 °C on MA and on tryptic soy agar (TSA; Oxoid/ThermoFisher Scientific) and observed after 3 and 5 days. Growth at different temperatures was tested at 4, 9, 21, 25, 30, 37, 40 and 44 °C on MA and in MB. For the latter, growth was assessed by measuring optical density at OD_600nm_. The following tests were performed on strain APC 4016^T^ and, unless stated otherwise, were carried out aerobically at 30 °C. Anaerobic growth was assessed on MA incubated in an AnaeroGen anaerobic system (Oxoid) for up to 14 days. The NaCl range for growth was tested in MB with the NaCl concentration adjusted to within the range 0–22 % w/v (in increments of 1 % from 0–10 % NaCl, and in increments of 2 % from 12–22 % NaCl). The pH range for growth was tested in MB from pH 4 to 12 (in increments of 0.5 from pH 4 to 9 and increments of 1 from pH 9 to 11). The pH was adjusted by the addition of 1 M HCl and/or 1 M NaOH. Growth was assessed by measuring optical density at OD_600nm_ daily for 4 days. Cells were examined using phase contrast microscopy and scanning electron microscopy (SEM). SEM imaging was carried out by UCD Conway Imaging Core (UCD Conway Institute of Biomolecular and Biomedical Research, University College Dublin, Ireland). Gram staining was carried out according to standard methods. Production of oxidase was tested using oxidase strips (Millipore) according to the manufacturer’s instructions. Production of catalase was tested by transferring a mass of colonies onto a glass slide and exposing them to 1–2 drops of 3 % hydrogen peroxide and observing for effervescence. Observation for motility was carried out using the hanging drop method according to the method outlined by Bernardet *et al*. [47] except that a cavity microscope slide was used. The presence of flexirubin-type pigments was tested using the KOH method as described by Bernardet *et al.* [47]. Production of hydrogen sulphide was determined using Watman lead acetate strips (Sigma-Aldrich) suspended above an inoculum of the strain in MB in a test tube and was monitored for up to 7 days.

Artificial seawater (ASW) salts were prepared for the following assays according to Kurilenko *et al.* [48] as follows: 30 g l^−1^ NaCl, 5.94 g l^−1^ MgSO_4_·7H_2_O, 4.53 g l^−1^ MgCl_2_ ·6H_2_O, 0.64 g l^−1^ KCl and 1.3 g l^−1^ CaCl_2_·2H_2_O. Assimilation of Tween 80 and starch was tested by plating on agar medium (15 g l^−1^ agar) containing ASW salts, 5 g l^−1^ peptone, 1 g l^−1^ yeast extract, 0.1 g l^−1^ K_2_HPO_4_, and 1 % v/v Tween 80 or 0.2 % w/v starch. Hydrolysis of DNA was determined using DNase Test Agar (Thermo Scientific) supplemented with ASW salts. Hydrolysis of casein was tested on agar medium containing ASW salts supplemented with 10 % w/v skimmed milk powder. Hydrolysis of cellulose was determined by culturing APC 4016^T^ on agar medium containing ASW salts, 0.2 % w/v cellulose and 0.5 % w/v peptone; following incubation the plate was then stained with 1 % Congo red, washed with 1 M NaCl and observed for zones of clearance around colonies.

Enzyme activity was assessed using the API ZYM (bioMérieux) test kit which was incubated at 30 °C for 18 h. Other biochemical tests were carried out using the API 20E, API 20NE kits (bioMérieux) and the GENIII MicroPlate (Biolog). Cells were prepared in 0.85 % NaCl for the API kits. For the GENIII MicroPlate, protocol B was used with inoculating fluid B supplemented with 2 % NaCl. The results were recorded daily for up to 4 days. Antibiotic susceptibility was determined using the disc-diffusion method [49]. Muller–Hinton agar (Oxoid) was used with commercial antibiotic discs (Oxoid) with the following antibiotics: ampicillin (10 µg), chloramphenicol (30 µg) erythromycin (15 µg), gentamicin (10 µg), kanamycin (30 µg), lincomycin (15 µg), neomycin (30 µg), novobiocin (5 µg), oleandomycin (15 µg), penicillin G (10 U), polymyxin B (300 U), rifampcin (30 µg), streptomycin (10 µg) and tetracycline (30 µg). Zones of inhibition were measured after 36 h at 30 °C. However, due to the lack of CLSI breakpoint data for interpretation criteria for the genus *Planococcus*, antibiotic susceptibility and resistance were interpreted as no growth or growth, respectively.

Analyses of cellular fatty acids, polar lipids and respiratory quinones was carried out by DSMZ (Leibniz Institute DSMZ–German Collection of Microorganisms and Cell Cultures GmbH, Braunschweig, Germany). For fatty acid and polar lipid analyses, strain APC 4016^T^ was prepared by culturing on MA at 25 °C. Cellular fatty acids were determined using the Sherlock MIS (midi) system (version 6.1; TSBA40 method, TSBA6 calculation).

## Results

### Genome features and characteristics

**Fig. 1.**
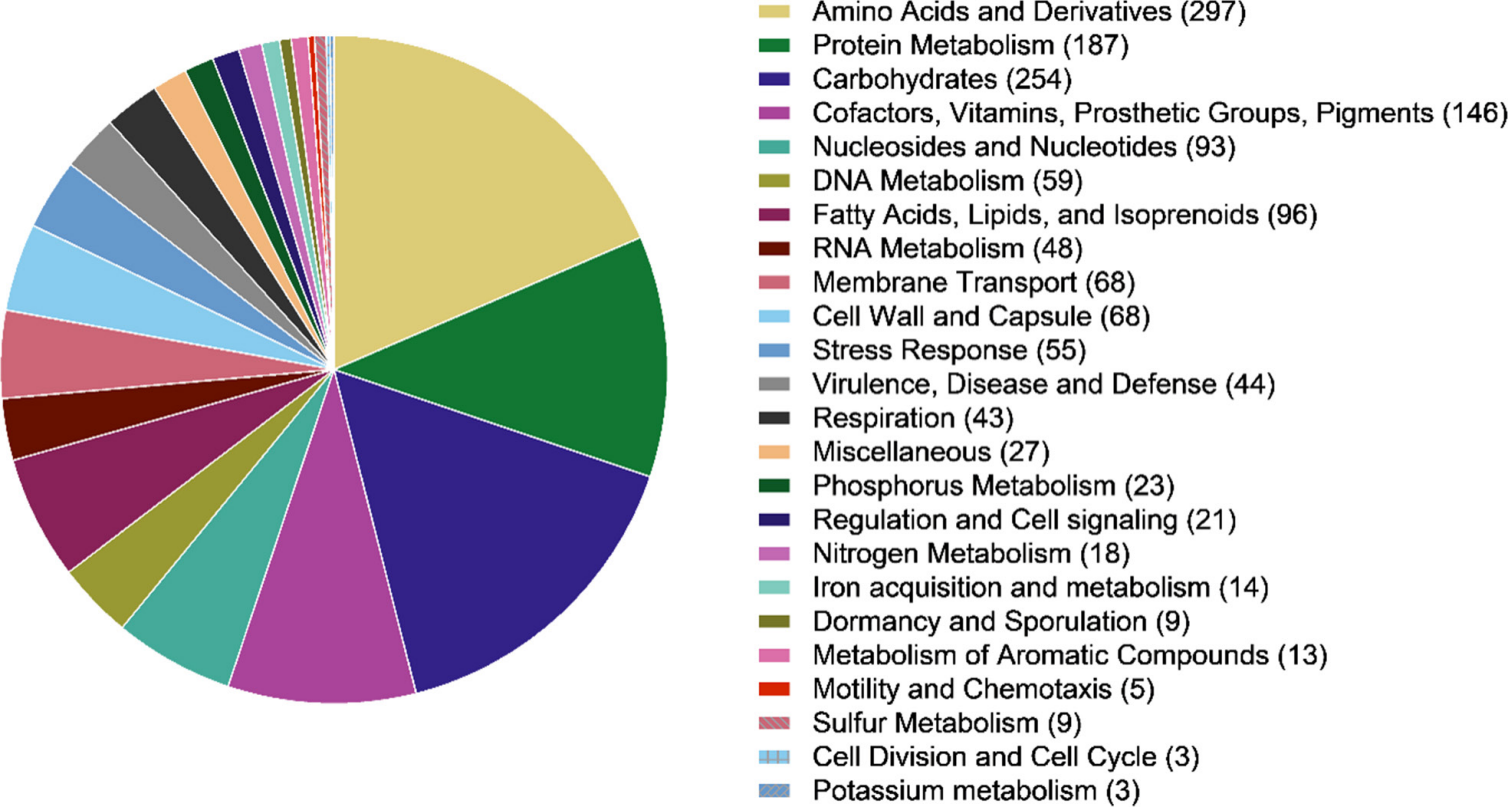
Overview of the subsystem features of the draft genome of strain APC 4016^T^ based on the rast annotation and seed database. The counts of features per subsystem category are given. The total number of subsystem features was 1603.

The draft genome assembly of strain APC 4016^T^ was 3 640 984 bp in length with a G+C content of 45.25 mol%. An overview of the general characteristics of the draft genome is given in Table 1. Using rast, 1603 subsystem features were identified across 24 subsystem categories. An overview of the composition of rast subsystem features is shown in Fig. 1. The largest subsystem categories were dedicated to ‘amino acids and derivatives’, at 297 features/genes (18.5 % of the total), followed by ‘carbohydrates’, at 254 features (15.8 %) and ‘protein metabolism’ at 187 features (11.7 %). Three secondary metabolite BGCs were identified using antiSMASH, encoding two terpenes, one of which shared 28 % similarity to the BGC of carotenoid, and a type-3 polyketide synthase (T3PKS) which shared 8 % similarity to beta-lactam. A BGC encoding two lanthipeptides (class II/LanM-associated) was detected using bagel4, the biosynthetic genes of which shared 50 and 55.6 % match with cerecidin (*Bacillus cereus*), respectively (data from Uniacke-Lowe *et al.* [29]). An overview of the antiSMASH and bagel screening results is given in Table S1.

**Table 1.** General characteristics of the draft genome of strain APC 4016^T^

Genomic feature	
Draft genome size (bp)	3 640 984
G+C content (mol%)	45.25
N50 (kb)	727.4
L50 (kb)	2
Number of contigs	21
Protein-coding genes (CDS)	3614
rRNA genes	5
tRNA genes	40
tmRNA	1

### 16S rRNA gene and genome phylogenetic analysis

**Fig. 2.**
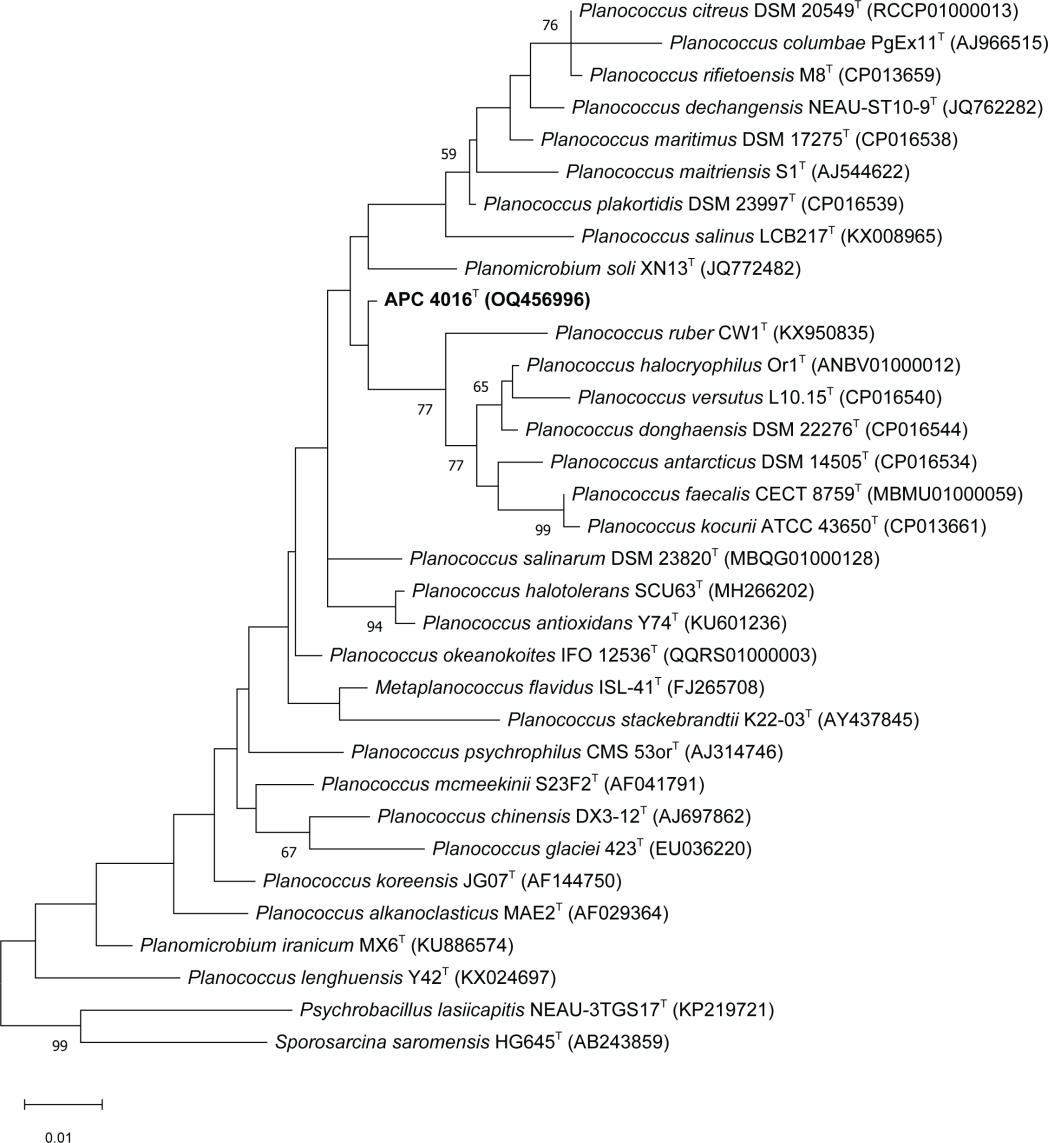
Maximum-likelihood evolutionary tree of distances based on 16S rRNA gene sequences, showing the relationship of strain APC 4016^T^ and related species. Bootstrap values based on 1000 replicates (>50 %) are shown. The tree was rooted by 16S rRNA gene sequences from *Psychrobacillus lasiicapitis* NEAU-3TGS17 and *Sporosarcina saromensis* HG645 of the family *Caryophanaceae*.

The 16S rRNA gene sequence of strain APC 4016^T^ is 1548 bp in length. Analysis of the 16S rRNA gene sequence using the EzBioCloud search tool indicated that APC 4016^T^ was most closely related to members of the genus *Planococcus* (*Planococcus*/*Planomicrobium*) within the family *Caryophanaceae*. The 16S rRNA gene sequence of APC 4016^T^ showed the highest similarity to *Planomicrobium okeanokoites* IFO 12536^T^ (98.98 % similarity) followed by *Planococcus plakortidis* DSM 23997^T^ (98.64 %), *Planococcus salinarum* DSM 23820^T^ (=ISL-16^T^, 98.64 %), *Planococcus donghaensis* DSM 22276^T^ (98.57 %) and *Planococcus halotolerans* SCU63^T^ (98.52 %). Completeness values were all 100 %. These strains were chosen as reference strains for the comparative phenotypic and chemotaxonomic analyses. The maximum-likelihood phylogenetic tree created from the 16S rRNA gene sequences (Fig. 2) shows the evolutionary relatedness between APC 4016^T^ and the EzBioCloud hits. There was slight variability in topology between the maximum-likelihood, neighbour-joining (Fig. S1, available in the online version of this article) and maximum-parsimony (Fig. S2) trees, likely due to low bootstrap confidence. However, in all three trees, APC 4016^T^ either formed its own clade or clustered with *P. okeanokoites* IFO 12536^T^.

A summary of the overall genome relatedness between APC 4016^T^ and the reference strains is given in Table 2. The pairwise ANI values between APC 4016^T^ and the reference *Planococcus*/*Planomicrobium* genomes ranged from 82.95 to 83.76 %. The ANI coverage ranged from 6.2 to 52.7 %. The corresponding graphical output of ANI pairwise values from pyani is shown in Fig. S3. The dDDH (d4) values ranged from 19.5 to 22.2 %. These ANI and dDDH values are well below the general thresholds for taxonomic species of 95–96 and 70 %, respectively [50]. These values are especially important to consider when the 16S rRNA gene sequence % identity is ≥98.7 % [50]. The maximum-likelihood tree generated from the roary core gene (*n*=67) alignments is shown in Fig. S4. This tree shows strain APC 4016^T^ clusters closely to a clade formed by *Planomicrobium stackebrandtii* K22-03^T^, *Planococcus antarcticus* DSM 14505^T^, *Planococcus faecalis* AJ003^T^, *Planococcus kocurii* ATCC 43850^T^, *Planococcus versutus* L10 15^T^, *P. donghaensis* DSM 22276^T^ and *Planococcus halocryophilus* DSM 24743^T^. The position of APC 4016^T^ within this tree indicates that it is a separate species among these strains. These results show that although APC 4016^T^ shared a high 16S rRNA gene sequence % identity to the reference strains, their genomes were not highly similar.

**Table 2.** Summary of genomic comparisons between APC 4016^T^ and closely related type strains dDDH, digital DNA–DNA hybridization; ANI, average nucleotide identity; coverage, ANI alignment coverage.

Query strain	Subject strain	16S ID (%)	dDDH (d4)	ΔG+C (mol%)	ANI (%)	Coverage (%)
APC 4016^T^	*Planococcus okeanokoites* IFO 12536^T^	98.98	20.1	5.9	83.8	8.7
	*Planococcus plakortidis* DSM 23997^T^	98.64	19.5	4.7	82.9	6.9
	*Planococcus donghaensis* DSM 22276^T^	98.64	22.2	5.2	83.2	27.7
	*Planococcus salinarum* ISL-16^T^	98.64	19.8	1.54	83.8	7.7
	*Planococcus halotolerans* SCU63^T^	98.57	19.9	0.7	83.7	8.8

### Cell morphology, biochemical and phenotypic characterization

**Fig. 3.**
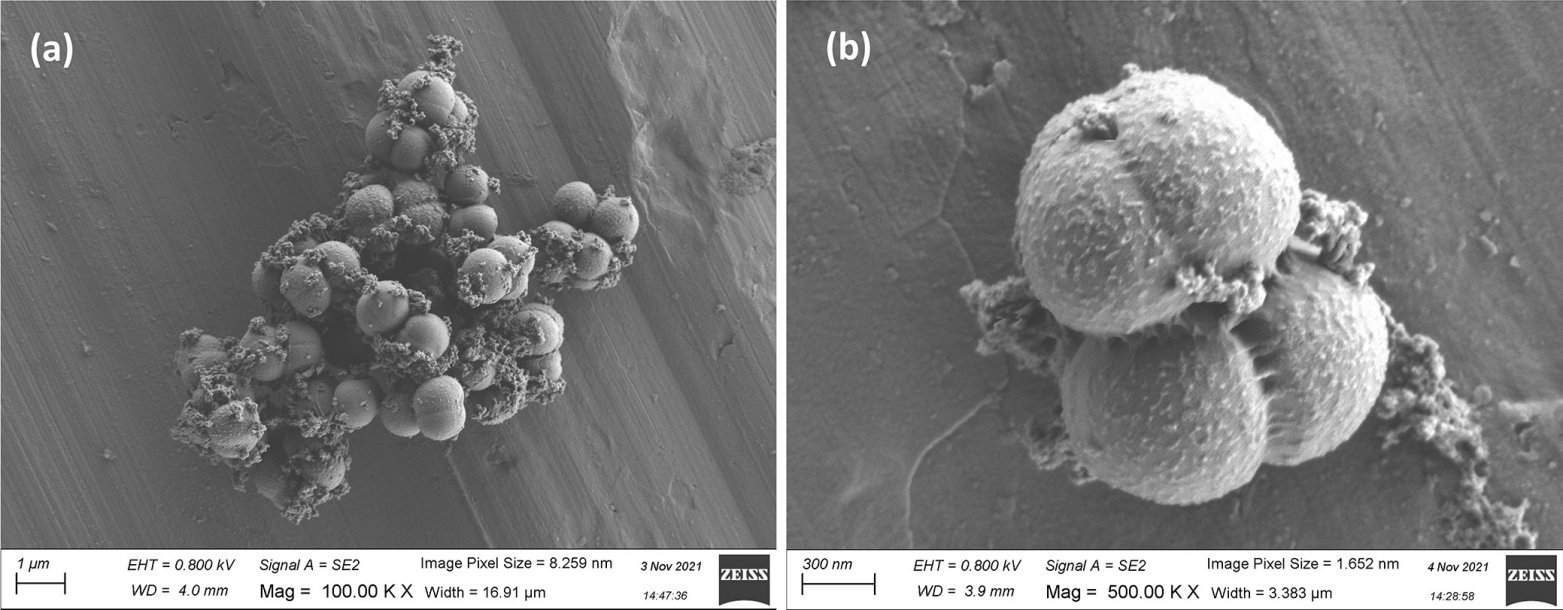
Scanning electron microscopy image of cells of APC 4016^T^ at ×100 K (**a**) and ×500 K (**b**).

**Fig. 4.**
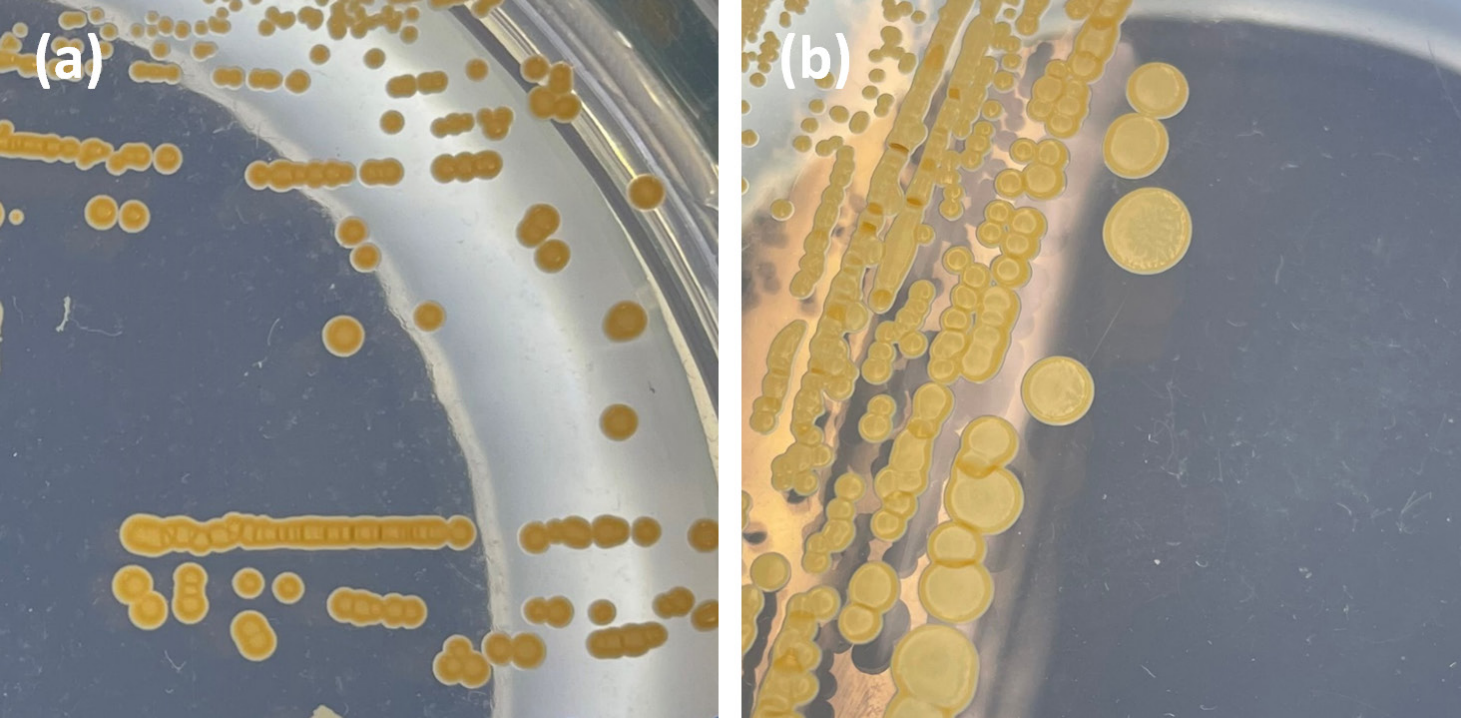
Colonies of APC 4016^T^ after culturing at 25 °C for 5 days on marine agar (**a**) and tryptic soy agar (**b**).

Cells of strain APC 4016^T^ are aerobic, motile cocci, 0.6–0.9 µm in diameter, occur in clusters of two to eight (Fig. 3) and are Gram-stain positive to variable (Fig. S5). Motility was observed as tumbling and spinning motions. After culturing on MA for 3 days, colonies of strain APC 4016^T^ appeared smooth circular and light orange with darker coloured edges, and 1–1.2 mm in diameter. Colonies cultured on TSA were paler in colour and slightly crateriform (Fig. 4). Flagella were not observed in SEM imaging (Fig. 3). The colony morphology after 5 days of incubation is shown in Fig. 4. From the API ZYM assay, strain APC 4016^T^ was positive for alkaline phosphatase, esterase, esterase lipase, leucine arylamidase, α-chymotrypsin, naphthol-AS-BI-phosphohydrolase and α-glucosidase activity; weakly positive for valine arylamidase, cystine arylamidase, acid phosphatase and β-galactosidase, and negative for lipase, trypsin, α-galactosidase, β-glucuronidase, β-glucosidase, mannosidase and fucosidase activity. Strain APC 4016^T^ was susceptible to all of the tested antibiotics: ampicillin (10 µg), chloramphenicol (30 µg), erythromycin (15 µg), gentamicin (10 µg), kanamycin (30 µg), lincomycin (15 µg), neomycin (30 µg), novobiocin (5 µg), oleandomycin (15 µg), penicillin G (10 U), polymyxin B (300 U), rifampcin (30 µg), streptomycin (10 µg) and tetracycline (30 µg). The antibiotic susceptibility profile of strain 4016^T^ with corresponding zone of inhibition measurements is given in Table S2. Due to the lack of CLSI guidelines and breakpoint data for the genus *Planococcus*, susceptibility and resistance were interpreted as no growth or growth. We also note the lack of standardized antibiotic susceptibility testing methods for members of this genus in the literature, such as the use of alternative media instead of Mueller–Hinton, and varying growth temperatures. This represents a deficiency in the standardization within this group; however, we have reported our results in line with existing literature.

Differential characteristics between APC 4016^T^ and the reference *Planococcus*/*Planomicrobium* strains are provided in Table 3. All the data for strain APC 4016^T^ is from this study, the data for the reference strains was acquired from the respective reference papers referred to in the Table 3 description. In the phenotypic assays, APC 4016^T^ shared a number of characteristics that are in accordance with members of the genus *Planococcus*, such as positive for catalase, negative for urease and hydrolysis of starch and a predominance of menaquinones 7 and 8 as the cellular respiratory quinones [2]. However, strain APC 4016^T^ differed in that it was positive for nitrate reduction and hydrolysis of Tween 80, weakly positive for hydrolysis of aesculin and contained menaquinones MK5 and MK6, as well as MK7 and MK8. Strain APC 4016^T^ demonstrated a relatively wide temperature range for growth of 4–40 °C, similar to the reference strains (Table 3). Strain APC 4016^T^ also demonstrated relatively wide %NaCl and pH ranges for growth of 0–12 % (optimum 1 %) and pH 5.5–12 (inclusive; optimum, pH 7–7.5), respectively. Growth above pH 12 could not be determined due to instability of the growth media at higher pH. In contrast to the majority of the closely related reference strains, APC 4016^T^ was negative for oxidase and hydrolysis of casein but positive for β-galactosidase.

**Table 3.** Differential characteristics of APC 4016^T^ and the reference *Planococcus*/*Planomicrobium* type strains Strains: 1, APC 4016^T^ (data from this study); 2, *P. okeanokoites* IFO 12536^T^ (data from [2,4] and [23]) ; 3, *P. plakortidis* DSM 2399^T^ (data from [51]); 4, *P. salinarum* DSM 23820^T^ (data from [3,52]); 5, *P. donghaensis* DSM 22276^T^ (data from [15]); 6, *P. halotolerans* SCU63^T^ (data from [53]). +, Positive; −, negative; w, weakly positive; nd, not determined; V, variable.

Characteristic	1	2	3	4	5	6
Cell shape	Coccoid	Rods	Coccoid	Coccoid, short rods or rods	Coccoid	Coccoid, short rod
Gram stain	+ to V	+ to V	+	+	+	+
Colony colour	Light orange	Bright yellow/ orange	Dull yellow/orange	Pale yellow	Orange	Moderate orange
Motility	+	+	+	−	+	+
Range for growth:						
NaCl (% w/v)	0–12	0–7	2–7	0–13	0–12	15
pH	5.5–12.0	nd	6–10	>6*	>7*	6.5–9.0
Temperature (°C)	4–40	20–37†	15–37	4–38	4–37	0–40
Oxidase	−	w	+	+	+	+
Nitrate reduction	+	−	−	−	nd	−
Hydrolysis of:						
Tween 80	+	−	nd	+	−	−
Casein	−	+	−	+	+	+
Gelatin	w	+	−	−	−	−
Starch	−	−	−	−	+	−
Aesculin	w	−	−	−	+	nd
Utilization of:						
d-Glucose	−	−	−	−	+	+
Maltose	−	−	−	−	nd	+
d-Mannitol	−	−	−	−	+	+
d-Mannose	−	−	−	−	−	+
Raffinose	−	−	+	−	nd	nd
Sucrose	−	−	−	−	+	+
Acid production from:						
Glucose	−	−	+	−	−	nd
Enzyme activity:						
β-Galactosidase	+	nd	−	−	+	−
Predominant menaquinones	MK-5, MK-6, MK-7, MK-8	MK-7, MK-8	MK-6, MK-7, MK-8	MK-7, MK-8	MK-7, MK-8	MK-7, MK-8

*Only minimum value for growth reported.

†Only optimum range reported.

The major (>10 % of total) cellular fatty acids of strain APC 4016^T^ were iso-C_14 : 0_ (12.5 %), anteiso-C_15 : 0_ (38.9 %), and C_16 : 1_ ω-Alc. (19.2%). The overall cellular fatty acid composition profiles of strain APC 4016^T^ and the reference *Planococcus*/*Planomicrobium* strains were similar (Table 4). Strain APC 4016^T^ differed slightly in that it had very few straight-chain fatty acids and did not contain iso-C_16 : 0_ as a major component (>10 %) but contained a significant proportion of a unique summed feature, iso-C_17 : 1_ I/anteiso B (which could not be distinguished by the midi system). The cellular polar lipids of strain APC 4016^T^ were phosphatidylglycerol, diphosphatidylglycerol, phosphatidylethanolamine and three unknown lipids (Fig. S6).

**Table 4.** Cellular fatty acid profiles of APC 4016^T^ and the reference *Planococcus*/*Planomicrobium* type strains Strains: 1, APC 4016^T^ (data from this study); 2, *P*. *okeanokoites* IFO 12536^T^ (data from [4] and [54]); 3, *P*. *plakortidis* DSM 2399^T^ (data from [51]); 4, *P*. *salinarum* DSM 23820^T^ (data from [51]); 5, *P*. *donghaensis* DSM 22276^T^ (data from [15]); 6, *P*. *halotolerans* SCU63^T^ (data from [53]). –, Not detected; tr, trace amount (<1 %).

Fatty acid	1*	2†	3*	4*	5*	6‡
Straight chain:						
C_12 : 0_	–	–	–	9.6	–	–
C_14 : 0_	–	–	1.5	5.5	–	–
C_15 : 0_	–	–	3.3	–	1.7	–
C_16 : 0_	tr	1.1–2.2	1.9	4.0	6.3	6.3
C_17 : 0_	1.1	–	4.0	–	1.3	3.7
C_18 : 0_	–	–	tr	7.4	–	1.8
Branched:						
iso-C_14 : 0_	**12.5**	**39.1–40.4**	9.6	5.0	3.4	**10.0**
iso-C_15 : 0_	2.9	0–1.1	**13.2**	–	4.7	5.4
anteiso-C_15 : 0_	**38.9**	3.2–4.0	**34.2**	**35.1**	**43.8**	**39.7**
iso-C_16 : 0_	6.3	**24.5–29.0**	**11.7**	**11.8**	9.4	**10.6**
iso-C_17 : 0_	1.4	–	2.9	–	6.4	–
anteiso-C_17 : 0_	2.3	–	5.5	7.7	**15.5**	5.6
iso-C_17 : 1_ ω10*c*	2.7	–	–	–	tr	–
iso-C_18 : 0_	1.1	tr	1.9	–	tr	tr
Unsaturated:						
C_16 : 1_ ω7*c*-alcohol	**19.2**	**17.3–28.1**	7.0	**10.8**	1.6	**12.9**
C_16 : 1_ ω11*c*	2.4	2.1–5.4	tr	–	1.3	–
C_18 : 1_ ω9*c*	–	0–1.0	–	–	–	2.7
Summed feature 4:						
iso-C_17 : 1_ I/anteiso B	8.7	–	–	–	–	–
Summed feature 5:						
iso-C_17 : 1_ I and/or anteiso-C_17 : 1_ B	–	–	1.2	–	1.9	–

* Analysis carried out at 25 °C.

† Analysis carried out at 28 °C.

‡ Analysis carried out at 29 °C.

Based on the combination of phylogenetic distance, phenotypic and biochemical characteristics, strain APC 4016^T^ represents a novel species for which we propose the name *Planococcus notacanthi* sp. nov.

## Description of *Planococcus notacanthi* sp. nov.

*Planococcus notacanthi* (not.a.can’thi. N.L. gen. n., *notacanthi,* pertaining to the fish it was isolated from, *Notacanthus chemnitzii*).

Cells are aerobic, motile cocci, 0.6–0.9 µm in diameter (arranged in clusters of 2 to 8), Gram-stain positive to variable. Colonies are light orange, circular, smooth on MA and slightly crateriform on TSA, raised, 1–1.2 mm in diameter. Catalase positive and oxidase negative. Flexirubin-type pigments are not detected. The temperature range for growth of is 4–40 °C (optimum, 25–28 °C). The NaCl range for growth is 0–12 % (optimum 1 %). The pH range for growth is pH 5.5–12 (optimum, pH 7–7.5). Positive for hydrolysis of DNA and Tween 80. Negative for hydrolysis of agar, casein, cellulose and starch. Negative for production of H_2_S. From API 20E: positive for β-galactosidase and weakly positive for tryptophan deaminase activity. Negative for arginine dihydrolase, lysine decarboxylase, ornithine decarboxylase, urease, citrate utilization, indole, acetoin, gelatinase and H_2_S production. Negative for fermentation of/acid production from glucose, mannitol, inositol, sorbitol, rhamnose, sucrose, melibiose, amygdalin and arabinose. From API 20NE: positive for nitrate reduction, production of β-galactosidase and weakly positive for hydrolysis of aesculin; negative for production of indole, urease, arginine dihydrolase, hydrolysis of gelatin and assimilation of d-glucose, l-arabinose, d-mannose, d-mannitol, *N*-acetyl-glucosamine, maltose, potassium gluconate, capric acid, adipic acid, malic acid, trisodium citrate and phenylacetic acid. From Biolog GENIII: Positive for oxidation of dextrin, l-fucose, d-glucose-6-PO_4_, d-fructose-6-PO_4_, d-glucuronic acid, glucuronamide, quinic acid, l-lactic acid, α-keto-glutaric acid, acetoacetic acid and acetic acid, and weakly positive for d-fucose, d-galacturonic acid and l-galacturonic acid. Major cellular fatty acids are iso-C_14 : 0_, anteiso-C_15 : 0_, and C_16 : 1_ ω-Alc. Respiratory quinones are MK5, MK6, MK7 and MK8. Cellular polar lipids are phosphatidylglycerol, diphosphatidylglycerol, phosphatidylethanolamine and three unknown lipids.

The type strain is APC 4016^T^ (=DSM 115753^T^=NCIMB 15463^T^), isolated from the skin of a deep-sea snub-nosed spiny eel, *Notacanthus chemnitzii*, from a depth of 1000 m in the Northwest Atlantic Ocean (43.282 N 49.121 W). The genome of the type strain is 3.6 Mb with a DNA G+C content of 45.25 mol%.

The GenBank accession numbers for the 16S rRNA gene sequence and the draft genome of strain APC 4016^T^ are OQ456996 and JASDCQ000000000, respectively.

## supplementary material

10.1099/ijsem.0.006298 Uncited Supplementary Material 1.
