# Supplementary material for: Planococcus notacanthi sp. nov., isolated from the skin of a deep-sea snub-nosed spiny eel
Source: Int J Syst Evol Microbiol. 2024 Mar 21;74(3):006298. doi: 10.1099/ijsem.0.006298 (PMC10963906; doi:10.1099/ijsem.0.006298)
Supplement: Uncited Supplementary Material 1. [file ijsem-74-06298-s001.pdf]

Supplementary Materials

Planococcus notacanthi sp. nov., isolated from the skin of a deep-sea snub-nosed spiny eel

Shona Uniacke-Lowe[1,2,3], Catherine Stanton[2,3], Colin Hill[1,2], Paul Ross[1,2]

[1]School of Microbiology, University College Cork, Ireland. [2]APC Microbiome Ireland, Cork, Ireland. [3]Teagasc Food Research Centre, Fermoy, Ireland.

* Correspondence: p.ross@ucc.ie

Table S1. Overview of the secondary metabolite BGCs and bacteriocin gene clusters encoded by strain APC 4016^T identified using antiSMASH and BAGEL, respectively.

Sample	Hit Type	Node	Most similar known hit / database hit	Similarity / Match (%)	Accession / Motif	Database
**APC 4016**	lanthipeptide class II	2	cerecidin	70	BGC0000502	antiSMASH
	terpene	2	carotenoid	100	BGC0000645	antiSMASH
	terpene	3	none	-	-	antiSMASH
	lanthipeptide class II x2	1	cerecidin	50,55.6	PF16934	BAGEL4

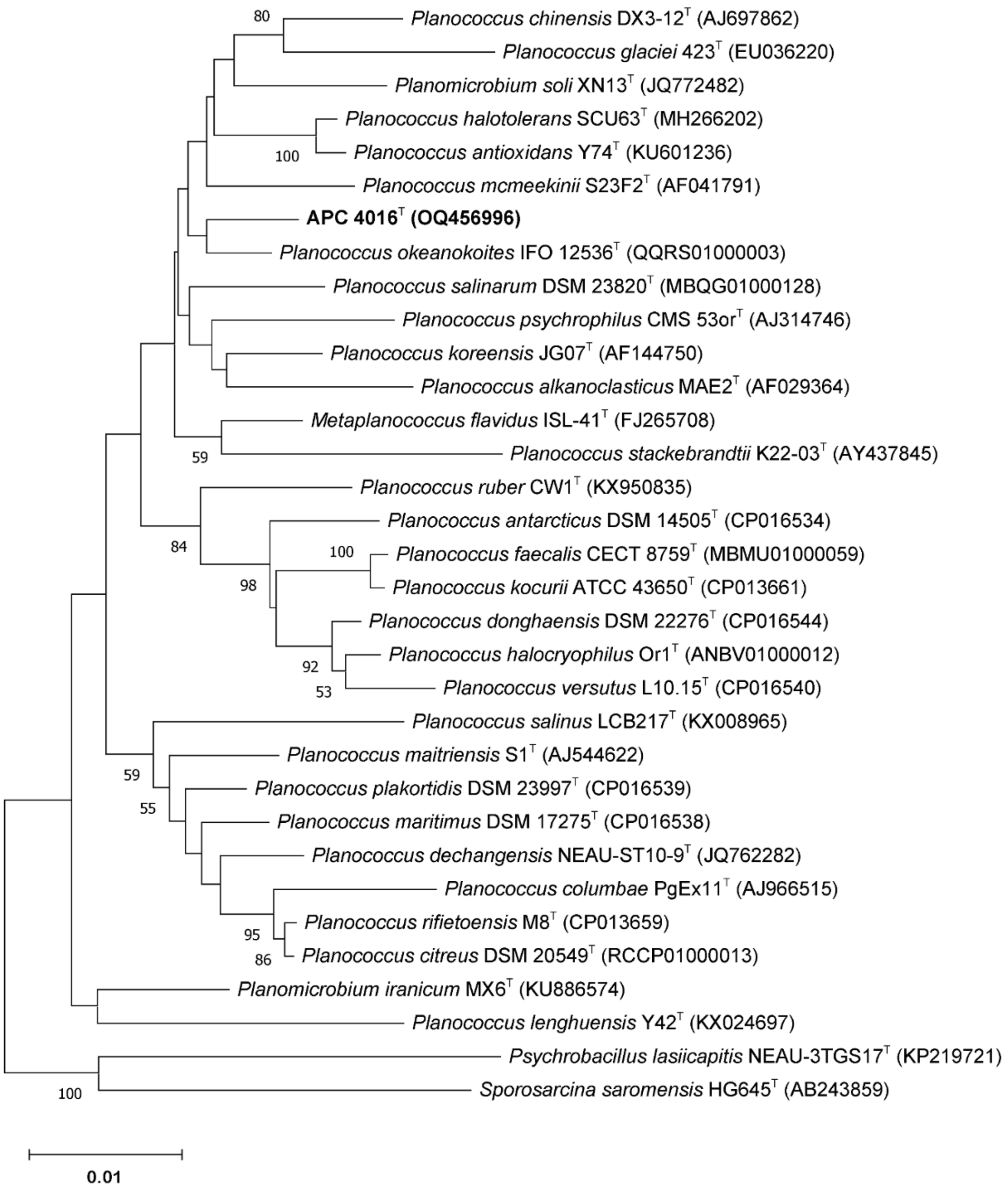

Fig. S1. Neighbor-joining tree of evolutionary tree of distances based on 16S rRNA gene sequences of APC 4016^T and related type strains. Bootstrap values based on 1000 replicates (>50%) are shown. The tree was rooted by 16S rRNA gene sequences from *Psychrobacillus lasiicapitis* NEAU-3TGS17 and *Sporosarcina saromensis* HG645 of the family *Caryophanaceae*.

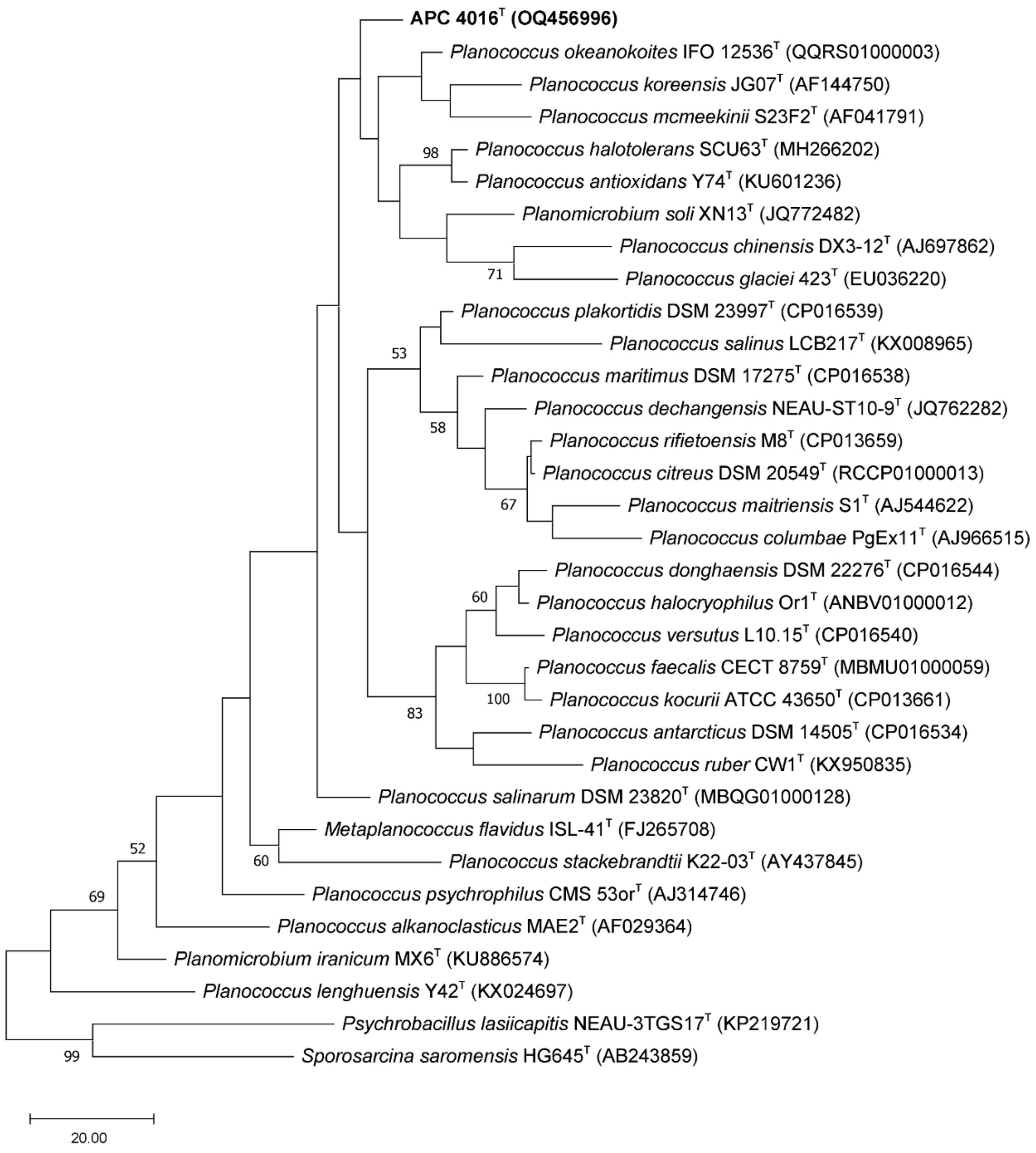

Fig. S2. Max-parsimony evolutionary tree of distances based on 16S rRNA gene sequences from APC 4016^{T} and related type strains. Bootstrap values based on 1000 replicates (>50%) are shown. The tree was rooted by 16S rRNA gene sequences from *Psychrobacillus lasiicapitis* NEAU-3TGS17^{T} and *Sporosarcina saromensis* HG645^{T} of the family *Caryophanaceae*.

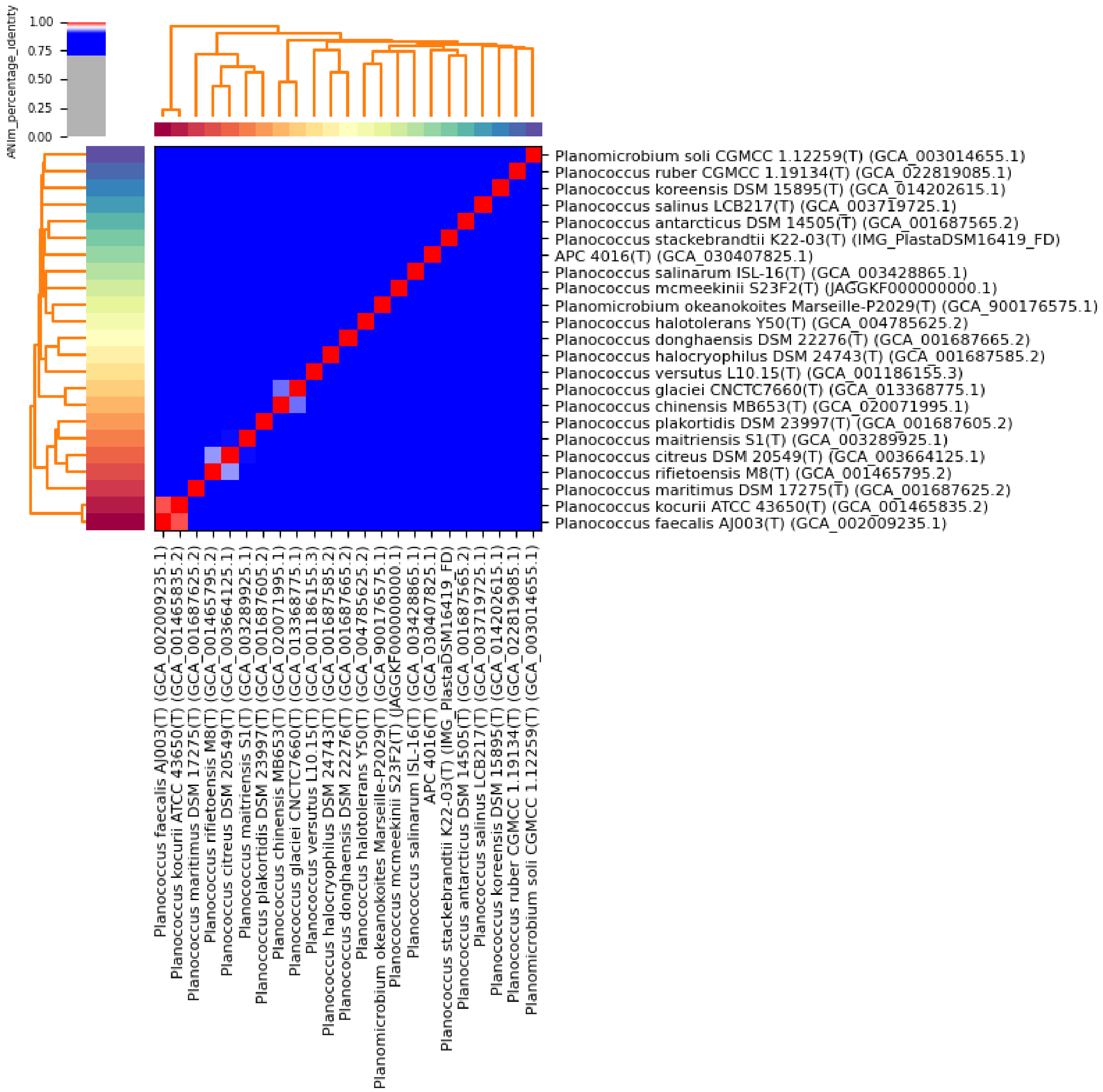

Fig. S3. Pairwise average nucleotide identity (ANI) values (as %) between APC 4016^T and all available *Planococcus*/*Planomicrobium* valid type strain genomes from the GenBank and JGI databases. ANI was calculated using PYANI and the MUMer method (ANIm). Blue cells correspond to ANI values < 95%, indicating separate species. Red cells correspond to ANI values >95%, indicating the same species. The colour intensities lighten as the ANI value approaches 95%. The dendrograms are created by hierarchal clustering of the pairwise ANI values.

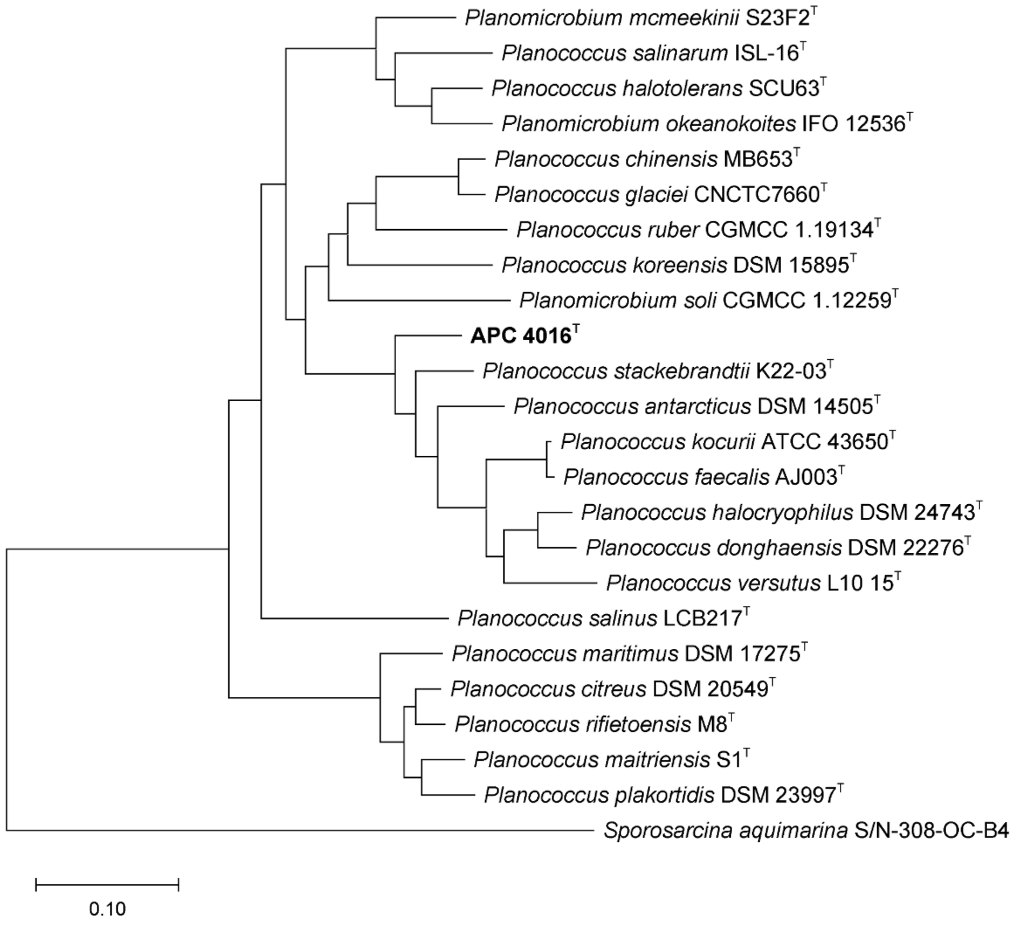

Fig. S4. Resulting maximum-likelihood phylogenetic tree created from the ROARY alignment of 67 core genes from APC 4016^{T} and *Planococcus*/*Planomicrobium* type strain reference genomes. *Sporosarcina aquimarina* S/N-308-OC-B4 was included as an outlier group.

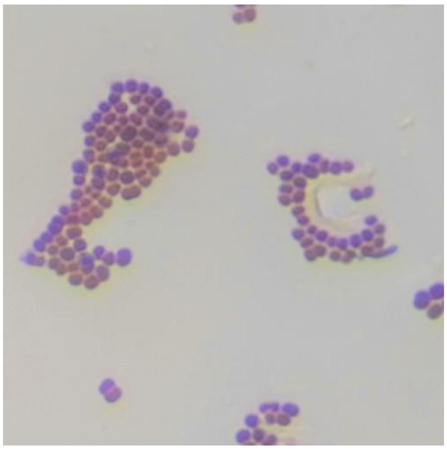

Fig. S5. Cells of strain APC 4016^T after Gram staining. Cells are Gram-positive to Gram-variable.

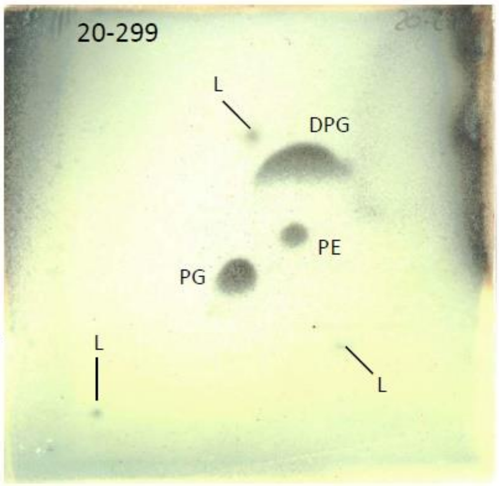

Fig. S6. Cellular polar lipid profile of strain APC 4016^{T} after separation by two-dimensional thin layer chromatograph.

L = lipid;

PE = phosphatidylethanolamine;

PG = phosphatidylglycerol;

DPG = diphosphatidylglycerol.

Table S2. Antibiotic susceptibility profile of strain 4016^T with corresponding zone of inhibition diameters (mm). Susceptibility and resistance were determined by no growth and growth, respectively.

Antibiotic	Disc content	Zone of inhibition (mm)	Result
ampicillin	10 µg	43 ±1.9	S
chloramphenicol	30 µg	33 ±2.5	S
erythromycin	15 µg	43 ±1.0	S
gentamicin	10 µg	20 ±1.6	S
kanamycin	30 µg	18 ±3.3	S
lincomycin	15 µg	10 ±4.0	S
neomycin	30 µg	17 ±5.4	S
novobiocin	5 µg	22 ±1.0	S
oleandomycin	15 µg	24 ±4.9	S
penicillin G	10 U	41 ±1.4	S
polymyxin B	300 U	20 ±0	S
rifampcin	30 µg	41 ±1.0	S
streptomycin	10 µg	16 ±2.6	S
tetracycline	30 µg	37 ±2.5	S

S = susceptible
